# *‘The mum has to live with the decision much more than the dad’*; a qualitative study of men’s perceptions of their influence on breastfeeding decision-making

**DOI:** 10.1186/s13006-018-0145-1

**Published:** 2018-01-16

**Authors:** Luke Hounsome, Sally Dowling

**Affiliations:** 1 Public Health England, Rivergate House, 2 Rivergate, Bristol, UK; 2 University of the West of England, Bristol, Glenside Campus, Blackberry Hill, Stapleton, Bristol, UK

**Keywords:** Breastfeeding, Fathers, Infant feeding, Decision-making, Qualitative

## Abstract

**Background:**

Although breastfeeding is widely acknowledged as the normal method of infant feeding, there are large variations in rates of initiation and duration. Several factors are linked to the likelihood of breastfeeding initiation, including the influence and opinion of the child’s father. There is limited research into men’s perception of their influence, or if they feel it appropriate to be involved in deciding how to feed their children. The aim of this study was to investigate, using a qualitative methodology, fathers’ perceptions of their influence on the decision to feed their child breastmilk or formula.

**Methods:**

Six men were recruited through Children’s Centres in Bristol, United Kingdom, and a phenomenological research methodology implemented using semi-structured interviews. Specific objectives were: to understand participants’ views on breastfeeding; understand if and how these views were discussed with their partner; to determine if participants believed involvement in the feeding decision is appropriate; to understand how they felt about the decision made; and to see if their views changed after the birth of their child.

**Results:**

Multiple themes emerged during analysis, including deferring of responsibility to the mother; breastfeeding as normal practice; change in attitude; involvement in parenting; and, advantages for the father. The men in the study accepted breastfeeding as normal behaviour, probably because of their upbringing in households where breastfeeding was practiced. There was consensus that women had more say in deciding to breastfeed, which was explained as a consequence of their greater involvement. It could also be interpreted as an unwillingness to interfere in an area perceived as ‘owned’ by women. Participants acknowledged that breastfeeding was more difficult than they had perceived.

**Conclusions:**

The key themes emerging from the interviews are suggestive of an impact on breastfeeding interventions that use the father as an intermediary. If they do not feel that they are ‘permitted’ to comment on their partner’s breastfeeding, then simply increasing knowledge of breastfeeding benefits in these men is likely to have minimal impact.

**Electronic supplementary material:**

The online version of this article (10.1186/s13006-018-0145-1) contains supplementary material, which is available to authorized users.

## Background

In developed countries, the main responsibility for making decisions about raising children, including how they are fed, has traditionally rested on the mother; often going hand-in-hand with their role as primary carer. For many, this includes the choice about whether to breastfeed or formula feed their babies. The introduction of easily-available contraception, laws on discrimination in the workplace, and the rise of feminism have all moved women and men towards an apparently more equal footing in many societies, although this is not experienced by all and not in all areas of their lives. There has been an increasing trend for fathers to be more involved with their children, and spend more time with them [1]. It is now expected in many relationships that men will be jointly involved in making decisions about their children. This is exemplified on a societal level in some countries by policy changes such as shared parental leave and pay, introduced in the United Kingdom (UK) in April 2015 [2] although the extent to which this is having any effect is, as yet, unclear [3].

There is a substantial body of literature which supports the value of breastfeeding in both developing and developed countries, recently confirmed and updated by the *Lancet Breastfeeding Series* papers [4]. The World Health Organization (WHO) recommends breastfeeding for the first two years of life; with exclusive breastfeeding for the first six months [5]. Breastfeeding initiation in the UK has been improving [6]; but the most recent data suggest that breastfeeding continuation has declined, with only half of women breastfeeding at two months [7]. Furthermore, in the UK there exists a large variation in breastfeeding rates between women in different socio-economic groups (with initiation rates in the ‘Professional’ occupations 16% higher than in ‘Routine’ occupations), of different ages, and of different ethnicities [6]. Such variation between groups has been described as social injustice [8] and is difficult to explain as the measurements of socio-economic status are intertwined with other factors, including employment [9], differing social norms [10] and concepts of self-efficacy [11]. The factors influencing non-initiation of breastfeeding, and early cessation, are therefore complex and overlapping.

Intention to breastfeed is a major step in the process of initiating breastfeeding. Application of models of health behaviour change (the theory of planned behaviour; the health action process approach; the transtheoretical model) to breastfeeding have confirmed that those who never plan to breastfeed are unlikely to do so [12]. However, personal experience also has a profound effect, perhaps explaining why, in one study, grandmothers’ own breastfeeding status was a stronger predictor of attitude than the mothers’ knowledge [13].

Although government and health agencies have made efforts to provide equitable access to services for breastfeeding support there is wide variation in breastfeeding rates between culturally separate groups [14] or adjacent city neighbourhoods [15]. Culture, religion and society all present different norms about how women and mothers should behave towards their children and partners [16]. These norms may account for the ethnic differences in breastfeeding initiation and duration which exist even when socio-economic differences are accounted for [17]. The perception of the breast as a sexual object [18] is particularly influential for younger mothers [19], who may feel pressure from partners to keep their breasts for sexual function only. Complex and conflicting societal messages around breastfeeding and breastmilk increases anxiety for women as these are simultaneously conceived as both pure and ‘dirty’ [20]. Cultural norms intersect with family opinions, and informal family support, which may explain why women who were breastfed are more likely to breastfeed their own children [21]. In many countries, there are first and second generation immigrants who may retain traditions and cultural norms from their home countries and societies [22]. These can dictate certain behaviour in relation to birth and breastfeeding [23]. Modification of these behaviours to ‘fit-in’ with the immigrant’s new country may decrease breastfeeding rates [24].

The perceived influence of the father has not been extensively studied, although it has been clearly shown that breastfeeding initiation and duration are linked to the father’s personal characteristics. For example, socioeconomic status and whether paternity leave was taken are significant predictors of Swedish men’s partners’ breastfeeding at 2, 4 and 6 months [25]. Men’s ethnicity, country of origin, education level, and socioeconomic status all contribute to different norms and expectations about breastfeeding [9] which may affect their attitude and ideas, and hence their influence on the feeding decision [26]. The attitude is critically important as women require willing support to breastfeed successfully. A survey of women who were breastfeeding at six months showed that 85% of them thought their husband or partner was supportive of breastfeeding [27]. Attitudes are variable, and are affected by ethnic group (although not necessarily with the same correlations as breastfeeding initiation) and by previous exposure to breastfeeding through siblings or other women. In two studies of men’s attitudes towards breastfeeding in the United States of America (US), one found them to have a neutral attitude overall [28] while another found the vast majority of men wanted their partner to breastfeed [29]. However, it may be the *perception* of the attitude which has the influence [30]. In the main, mothers acknowledge that they are influenced by their partner. However, it is of interest that some research has shown that influence is exerted unknowingly and that men may be influencing a woman’s decision to breastfeed without either of them being aware [26].

Because of the father’s influence in the breastfeeding decision there have been interventions in the UK, US and Australia aimed at men to try and increase breastfeeding rates. These have including the ‘debunking’ of myths around breastfeeding, and education on health benefits [31]. If the father feels he has no influence on the feeding decision, for whatever reason, such programmes will fail as there are not likely to be discussions or any other route of influence by which the fathers can pass on their knowledge.

The aim of this study was to investigate, using a qualitative methodology, fathers’ perceptions of their influence on the decision to feed their child breastmilk or formula. Specific objectives were: to understand participants’ views on breastfeeding; understand if and how these views were discussed with their partner; to determine if participants believe involvement in the feeding decision is appropriate; to understand how they felt about the decision made; and to see if their views changed after the birth of their child.

## Methods

We were interested in the lived experience of men, with regards to infant feeding decisions. A phenomenological methodology was chosen as the most appropriate to discover how men felt about their role in this important decision. In reviewing the phenomenological literature the first author was struck by van Manen’s comments on using personal experience as a starting point for research [32]. He was drawn to research about breastfeeding because of his experiences in talking about feeding for his own children, and a desire to understand what the experience of other men was. This also meant he did not believe he could bracket off these experiences as Husserl demands, and that an approach based on Heidegger’s philosophy was better suited [33].

Van Manen is one of the contemporary researchers who offers more specific methodological guidelines for phenomenological research [34]. His definition of description includes both the interpretive components of hermeneutics and the descriptive aspects of ‘pure’ phenomenology; the application of a phenomenological approach to this topic was therefore guided by the work of van Manen.

Study participants were recruited through local Children’s Centres. These are government funded organisations which offer family support; particularly in socially deprived areas. The need for men to be ‘recruited’ into research has been recognised [35]; we used Children’s Centres to approach a number of men with children in a reasonably short period of time. The first author approached all local Children’s Centres to speak to fathers’ groups about the research study. For those centres which did not run a fathers’ group, the first author attended other sessions to approach any men present, or talk to women about the study and ask them to pass on details to their partners. In the centres where women were approached at mixed groups, these women effectively became recruiting ‘agents’ and relied upon to pass information to their partners. However, they could also be perceived as gatekeepers, because if they did not want their partner to discuss their shared private life they could effectively prevent any involvement.

The sampling strategy was to sample for maximum variability of socio-economic background (based on the recruiting centre), ethnic origin, and number of children. These details were requested in the participant information sheet to allow sampling to take place. The intention of this sampling strategy was not to produce a generalizable result, but to hear as many different experiences as possible. The possibility that there would be a limited response to this study was considered and an opportunistic sampling strategy planned as a second strategy.

The main challenge to the successful implementation of this project was the recruitment of a sufficient number of men. Previous research has indicated an approach based on face-to-face recruitment would be most successful [35]. Therefore an important part of the project was to encourage as many Children’s Centres as possible to participate.

Six men were recruited; five through three children’s centres, with the sixth a friend of one of the men. Apart from the man who was passed information from a friend, all the men were recruited via their partners. This is consistent with the techniques of using a third party described by Oliffe and Mroz [35] who note that partners often influence or give permission to men to take part.

The experience of participants was captured using semi-structured interviews. This method is well-suited to situations where the field is not well understood as it allows the participants to talk about the experiences which are important to them and lead the discussion [36]. Interviews took place in the participant’s home, apart from one which was done at his place of work for convenience. They were guided by a series of questions (Additional file 1). Questions covered the participant’s awareness and knowledge of breastfeeding, their desired feeding plans, how the decision was made, and the outcomes. These were not necessarily asked in the same order for each interview, and in keeping with the concept of a semi-structured interview were mixed in with other questions pertinent at the time. However, this approach had to be balanced against concerns that male research participants do not always react well to non-specific questions [37]. Interviews were audio-recorded and later transcribed for analysis by the first author; both authors discussing emerging themes. All participants were anonymised (referred to here as Participants A-F) and any identifying information removed from transcripts.

NVivo software (QSR International, version 10) was used to manage the data. The process of analysis used was that outlined by Braun and Clarke [38]. Firstly, a general familiarity with the data was obtained by re-reading of the transcribed text. Then upon further reading, and using NVivo, the initial codes were generated. These codes were grouped into themes using an iterative process and reflecting on the ideas which naturally arose during the interviews process. Once the themes were named and defined, interpretation could be considered.

The participant information sheet described the aims of the study as well as the relevant points around consent, data protection and the right to withdraw from the study. As some research has shown men may not read this information [35], verbal checks were undertaken before the interview to ensure that all participants fully understood and consented.

Limitations included that, despite talking to a diverse range of people at the Children’s Centres, the participants for the study were a homogeneous group. All their children were initially breastfed and the majority continued for several months. All participants were White British and living with their partners. The experience of men from other cultural groups, those who live separately from the mother, and those who chose to not to breastfeed their children, are not represented in this sample. Similar studies have also tended to include participants who are from higher socio-economic groups [39, 40].

## Results

Six men were interviewed. All were living with their partners and had one or two children. All the children were under school age and so infant feeding decisions had, for these men, been a relatively recent topic. Five of the men were in paid employment, and one was a full-time parent. Although it was not specifically asked, it was possible for the lead author to infer that some of the men had degree-level education and were in professional careers.

Multiple themes emerged during analysis, including deferring of responsibility to the mother; breastfeeding as normal practice; change in attitude; involvement in parenting; and, advantages for the father. These are outlined and discussed below.

### Deferring of responsibility

The most prominent theme which emerged, discernible during the interview process itself, was the deferring of responsibility to the mother. The participants’ partners were the sole source of nutrition for their children and therefore they necessarily also had to deal with any accompanying challenges, including pain, interrupted sleep, and not having time away from the child. The men in this study recognised this:

*“With something like this it’s difficult I guess because there isn’t an equality in terms of the biology of it, so the mum has to live with the decision much more than the dad” [Participant E]*

*“It’s her boobs at the end of the day isn’t it? It requires an investment of a lot of time and energy from her” [Participant F]*

The participants talked about this inequality in terms of the decision on how to feed:

*“I think that our opinions are equally as valid but I think, as I’ve said already, that the actual decision would be . . . if [my partner] had wanted to stop breastfeeding there’s nothing I can do to change that decision.” [Participant B]*

*“But I remember also being really conscious that I wasn’t going to try and pressure [my partner] one way or the other, cause . . . I kind of felt like it wasn’t my place to dictate, I guess, what she does with her body.” [Participant F]*

The participants did not defer the decision in an attempt to avoid responsibility or because they had no desire to be involved, but because they felt it right that the power should lie with the mother. For one of the participants the deferring of the decision to the mother was associated also with the usual practice in the UK of the mother staying at home to look after the child in the period after birth:

*“Whoever is the full-time carer in the initial stages of . . . I guess after birth in those first few months, probably, and it’s usually the woman isn’t it, probably has more of a role in making those decisions, just basically because they’re around more” [Participant A]*

For feeding decisions after the birth which affected the mother more that the father, the decision was again framed as one over which the mother had greater say. In one case, the difficulties of feeding a second child while the first was still young had caused one couple to move to formula feeding:

*“. . . and with (my second child) although it wasn’t really my decision because the reasons we stopped breastfeeding were to do with [my partner] and how she was coping with it, to do with reasons of practicality” [Participant B]*

The converse case of deciding to stick with breastfeeding even when it was difficult was also credited to the women. This was often intertwined with the mother being more ideologically committed or determined to breastfeed:

*“She was very good at pushing through all that and staying true to what she thought was the best way for [my child] . . . it’s something she was very intent on doing” [Participant D]*

*“I remember it being difficult. But [my partner] [inaudible] through because she’s quite a determined woman when she sets her mind on doing something.” [Participant F]*

One participant mentioned the words ‘guilt’ and ‘guilty’ in relation to the determination to breastfeed and keep breastfeeding:

*“She would have been guilty and despondent that she wasn’t able to do it” [Participant D]*

*“It was definitely her main decision at the outset as to . . . and carrying on because she had to push through all the mastitis and pain involved in doing that, and sort of guilt at the outset that she couldn’t feed him enough” [Participant D]*

### Breastfeeding as normal practice

A further emerging theme was the consideration of participants that breastfeeding was a normal practice. Participants mostly had an awareness of breastfeeding from an early age:

*“Yeah, I’ve got two younger brothers so I imagine, certainly my youngest brother was being breastfed and I’ve got younger cousins and things. I couldn’t put an age on it . . .” [Participant B]*

*“Well I have a sister who’s two years younger than me. I guess I was aware that as soon as I’d stopped doing it that my younger sister, you know, as soon as she was born, was breastfed I think. Yeah I definitely remember her breastfeeding, so right from back then” [Participant E]*

However, there was no mention that this awareness had led to any particular opinion being formed about breastfeeding:

*“I definitely didn’t have any negative views towards it, or particularly strong feelings either way.” [Participant C]*

*“I certainly wasn’t disgusted by it, but I didn’t find it hugely interesting either” [Participant E]*

*“Not having any frame of reference or concern for it or even about it until [my partner] and I started to have [my child]” [Participant D]*

One participant did mention the lack of visibility of breastfeeding in the community he grew up in, but felt that this had little conscious effect on the decisions made:

*“And then as you got older, say teenage or young adult years, did you perceive breastfeeding as a normal thing that people did?” [Interviewer]*

*“No, but in the sense that, you know, obviously, you didn’t see it a lot – certainly not in the (area). Probably still don’t…didn’t see it around, didn’t see it being done” [Participant D]*

*“To a large extent what happened to either of us is not really relevant, although it may consciously or sub-consciously have affected one or both of our decision-making processes.” [Participant D]*

Although describing their experiences around breastfeeding as fairly neutral, there was an assumption for most participants that their children would be breastfed. One participant referred to this as ‘instinctive’, which can be interpreted as a state of believing an action is appropriate, without necessarily being able to detail exactly why:

*“I think there was . . . there was . . . a general assumption that they would be breastfed – probably . . . but there was no set plan or no real set ideas beyond that really.” [Participant A].*

*“I knew instinctively that I would try and support her to breastfeed.” [Participant F]*

As described in the literature review, knowledge about breastfeeding is a strong predictor of breastfeeding. Some of the participants expressed their knowledge of the benefits of breastfeeding for the child and mother:

*“I was also vaguely aware as well that there were certain, sort of health benefits to breastfeeding, if that was a course of action which could be pursued, in terms of development of immune system and suchlike” [Participant A]*

*“Because another thing I did know, as we’re talking about it, was that breastfeeding can be a good way of dealing with postnatal depression – and I remember hearing that in a TV show years ago” [Participant C]*

One participant was clear that he did not know, and had not attempted to look into it before the birth. When faced with the reality of the situation he started to investigate:

*“Did that prompt you to look yourself into any of the evidence around breastfeeding or become more educated on it, for want of a better word?” [Interviewer]*

*“Not to a great deal, but more so after [my child] arrived, because the whole thing, especially during the pregnancy bit, is a bit of an abstract concept . . . I didn’t know anywhere close to enough when we put it into practice” [Participant D]*

One participant felt that they had had a greater knowledge of breastfeeding than their partner:

*“And would you say your partner was equally well-informed?” [Interviewer]*

*“No. Not in a bad way, just . . . I tend to read more . . .” [Participant B]*

In this instance, although he felt that he had a better knowledge about breastfeeding, he would not share that with his partner. This links with the theme of deferring power to the partner on this topic. While not apparent from the literature, perhaps the men did not feel they could express knowledge or ideas on a subject which could be perceived as ‘owned’ by women:

*“Do you recall yourself telling her about it, because you knew?” [Interviewer]*

*“Yeah . . . that’s probably not something I would have done.” [Participant B]*

Knowledge was gained from a variety of sources. Some participants emphasised that benefits of attending organised groups:

*“. . . they obviously had a load of talks which were people who were trying to get you to come to the courses, and one of them was on breastfeeding. And that was really good and really interesting and it was done by a breastfeeding support group and I think that that probably did really persuade me to breastfeed [. . .] that definitely made me think ‘yeah we should give it a try’.” [Participant C]*

While for others it was health professionals:

*“We had a team of community midwives who were very supportive of the idea of breastfeeding.” [Participant A]*

*“So going through the whole pregnancy thing there’s quite a lot of breastfeeding propaganda basically [laughs] that’s thrown at you by the midwives . . . which I was quite convinced by, I’ve got a tendency to be quite trusting of professionals so if the doctor says so or the midwife says so I’ll probably believe them.” [Participant E]*

One participant did not feel that he could relate to the antenatal classes, because he felt different from the other men there. This limited the support or interaction he could get from other dads:

*“We went to one prenatal, antenatal, yeah, one of those classes things, and we were living . . . in [area] at the time and I felt like all the other dads were 10 years older than me and . . . I felt completely out of place and didn’t really talk to anyone and didn’t make any parental friends, so there wasn’t really anyone I would have talked about it with.” [Participant F]*

### Change in attitude

Some participants expressed a change in attitude due to their breastfeeding experiences. One had become much more pro-breastfeeding, partly because of a perception that his child had less illnesses than children of a similar age:

*“Yeah, I’d say I’ve just become more pro. I’ve always leant towards being pro-breastfeeding but in a . . . as I was saying earlier a bit apathetic in that fact that it didn’t really bother me either way.” [Participant C]*

*“. . . having witnessed that [my child] is five months old she’s not even really had a cough let alone any kind of serious illness; whereas people, friends of ours, who have bottle-fed their child, have just had a string of different ailments.” [Participant C]*

Others had realised that the choice of breast or formula-feeding was not necessarily a straightforward one. Their awareness of the difficulties that women might have while feeding was raised, and they had become more pragmatic. There was an understanding that if support was not there they too may have turned to formula-feeding:

*“I definitely think [my partner] is not a fan of formula milk basically . . . but when I say that, recognises that formula feeding comes largely because of, you know, people have to work, women have to work these days, people have to go back to work quickly.” [Participant A]*

*“I probably did change my attitude . . . I didn’t realise I think, just how . . . I guess I considered it a physical, mainly a physical or physiological process . . . but actually the social side of it, you need to learn, someone needs to teach you what angle to hold the head at – that’s not solely instinct is it?” [Participant F]*

Some men thought there was still some stigma attached to breastfeeding, particularly breastfeeding in public. They did not themselves believe that there was anything wrong with feeding in public, although may have had to adjust to the idea:

“*My own feeling is that, there’s sort of . . . there’s a set of, perhaps, stigmas about certain things that make breastfeeding difficult . . .” [Participant A]*

*“For me the bit I had to get over mentally was the stigma over breastfeeding in public and things like that and not being embarrassed about it.” [Participant C]*

Unlike public breastfeeding, some participants mentioned the concept of long-term feeding as abnormal:

*“Incidentally, one of the slight negative things that I had about breastfeeding was that my sister-in-law breastfed my niece until she was about two or three and I thought that’s going on a bit too long.” [Participant C]*

*“I do remember my best friend’s little brother breastfeeding until the age of four or five, which is unusually old, and it being considered weird by either my family, or somebody . . . making a comment about that being weird.” [Participant F]*

### Involvement with parenting

The participants were clear that in their minds it was a father’s place to be involved in child rearing and the decisions that accompany it. This may be a characteristic of the type of men who agreed to participate; men with higher educational achievement may be more likely to spend more time with their children [1, 41]:

*“I think that parenting should be a shared task, a shared responsibility, which means that both people who are involved should have a say.” [Participant B]*

*“It seems relatively obvious that having kids is a joint task, so there should be a joint approach towards it.” [Participant D]*

*“But as for fathers being involved I guess…yeah it’s something I didn’t really think about. I mean I think generally fathers should be involved in as many parenting decisions . . . and I think I’ve always thought that.” [Participant F]*

There was little recall of explicit discussions on decisions around breastfeeding. Some of the participants indicated that in the run-up to having children feeding decisions were perceived as less important than other child-related decisions:

*“It’s possible we had a conversation around that time as to how we wanted things to go but it was probably fairly low down the list of things we wanted to talk about in relation to babies.” [Participant B]*

*“I think that right from when they started talking about that in the early stages of going to see the midwife and doing antenatal classes I was pretty convinced that that was going to be the right thing to do. Uh, I don’t remember whether we had spoken about . . . whether me and [my partner] had spoken about that before or whether we’d started speaking about it then . . . I’m not really sure what order that came in, but yeah.” [Participant E]*

One participant had had conversations prompted by the experience of his sister:

*“We’d had the discussions around my sister-in-law breastfeeding . . . so my niece is nearly five, so what . . . started the debate was that my sister had my blood niece about two weeks before . . . (my partner’s) sister had her daughter, and they both breastfed but my sister stopped at six months because she went back to work, whereas (my partner’s) sister carried on for a lot longer. And we debated a lot on why you should breastfeed and for how long . . . most of it was when until, that was the debate rather than if you should.” [Participant C]*

Some participants had been more involved in discussions over weaning or other longer-term feeding-related decisions:

*“we ended up weaning him two weeks early – so not even early really – but we were . . . regularly talking through what we thought about that and whether we should switch over, how quickly and all those kind of things . . .” [Participant E]*

In previous research in the UK the decision to move to formula feed had been driven by men’s concern for their partner [42]. This was also the case with one participant in this study:

“*Yeah, we discussed it to the extent that [my partner] said, at some point I’m going to have to stop because this isn’t working. I think we kind of . . . I hope I didn’t put any pressure on her to continue any longer than she was able to but it was very much, not a choice as such but it had to be bottles or something was going to go quite badly wrong.” [Participant C]*

And other participants had considered the move because of the perceived inconveniences or difficulties of breastfeeding:

*“It was quite hard work when [my partner] first went back [. . .] and then I’d take [my second child] home to sleep. But if we weren’t there (my second child) would just wail, you know, and we did think about trying to give [my second child] a bottle at that point” [Participant A]*

*“So I think the only thing that would make me think not to breastfeed now is just the frustration around that [not taking a bottle of expressed milk], the baby becomes almost too dependent on the mother” [Participant C]*

### Advantages for the father

Some of the participants mentioned the advantages to men of breastfeeding, such as not having to wake in the night for feeding.

*“It seemed a decent way for me to get some sleep as well.” [Participant A]*

*“We know there’s some benefits for the mother as well…but there’s massive benefits for the dad, cause you can’t be collared for feeding the child in the middle of the night. It’s a ‘get out of jail free’ card to an extent.” [Participant F]*

*“I don’t know why dads haven’t cottoned on to this, and be more encouraging, as it’s a massive get-out if you can be not responsible for feeding.” [Participant F]*

## Discussion

Consistent with other studies, almost all participants said that they regarded their partner’s opinion as carrying more weight in decisions about breastfeeding initiation and duration [39, 40]. The participants did not defer the decision in an attempt to avoid responsibility or because they had no desire to be involved, but because they felt it right that the power should lie with the mother. For feeding decisions after the birth which affected the mother more that the father, the decision was again framed as one over which the mother had greater say.

One participant’s partner had experienced feelings of guilt when struggling to breastfeed, or would feel guilty if they had to stop breastfeeding. This may arise from breastfeeding being perceived as a normal or natural behaviour in the woman’s mind, and therefore any inability to fulfil this behaviour is ‘abnormal’ [39, 43]. Alternatively, the information (‘propaganda’ [Participant E]) around the benefits of breastfeeding may have been so firmly expressed to the women, that they feel they are letting down the health professionals who have supplied it, or indeed society which expects – or is perceived to expect – its members to follow the actions most advantageous to health [44].

All the men assumed their children would be breastfed. In a small study from the US, early awareness from growing up around breastfeeding was linked to intention to breastfeed [45]. This may be interpreted as being due to acceptance of the behaviour as normal because it was seen at an early age, as in the case of those participants who had seen siblings fed. Previous research has detailed that awareness, knowledge and contemplation can be prompted by encountering the behaviour [46] particularly in family or friends [47]. How though do we explain the seeming disinterest of the participants to breastfeeding before fatherhood? It is possible that the normalisation of breastfeeding linked to early childhood experience had led to a ‘shelving’ of the concept in teenage and young adult years in the same way we might have not particular thoughts about, such as walking or using cutlery.

Yet, when the appropriate time arises, i.e. impending parenthood, the concept re-emerges. A UK study has suggested that social norms and knowledge lead to an assumption of breastfeeding [39]. In the experiences described here it seemed that the combination of long-term awareness and belief that breastfeeding is a normal behaviour leads to the assumption that is will occur.

The participants had mixed knowledge of breastfeeding, and none mentioned any information or groups that were specifically tailored or targeted at the father. Some studies have suggested the need for specific information tailored to fathers-to-be [42]. The fact that there was generally some knowledge of the benefits of breastfeeding may have helped the men in this study be more comfortable in letting their partner make the decision, as the women were all intending to breastfeed. It may have been a different situation if the partner had wanted to formula-feed; although this is impossible to speculate on from this study.

The acceptance (albeit with a period of adjustment) of the participants about breastfeeding in public differs from a previous similar study. However, that study was conducted in the US with men from a more diverse cultural and ethnic background [40]. The views on ‘abnormal’ long-term breastfeeding are more consistent with societal opinion, which views feeding over the age of one year as unusual [48, 49], even though the WHO recommends it up to, and beyond, the age of two, alongside appropriate foods [5].

Although uncomfortable in being involved in breastfeeding decision-making, the participants were otherwise committed to shared parental decision-making. Greater involvement in discussions later in childhood may reflect the fact that the father is likely to be more affected by such decisions or involved in making them happen and therefore feels more able to take part or offer opinion; or it could possibly be because they have adjusted to a new experience and feel more qualified to comment. Previous work has highlighted that the father’s input into decision-making may be more valuable to the mother than they realise [42].

As far as we can see the advantages to men of breastfeeding are not discussed in the literature. There may be a certain embarrassment in society at voicing such an opinion as it might be viewed as selfish, or appearing that the father does not want to be involved with his children. It may just be reflective of the fact that men tend not to discuss infant feeding with other men.

## Conclusions

In this study we explored fathers’ perceptions of their involvement in infant feeding decisions. Using a phenomenological methodology and a thematic analysis several themes were identified as being important. The men interviewed all had good knowledge of breastfeeding, and were supportive. This did not, however, directly lead to any discussion about feeding decisions. Furthermore, some men reflected that their initial desire for their partner to breastfeed had been tempered by witnessing some challenges faced when they tried to breastfeed. The most strongly expressed feeling was that it was not appropriate for men to try to influence their partners in a decision which would not directly affect them. This is evidenced in the lack of discussion on feeding decisions before the birth; and the ‘pragmatic’ shift towards being more neutral on formula feeding. Whilst recognising that the men interviewed were not widely representative, these findings may have some bearing on practice if breastfeeding interventions are intended solely to improve men’s breastfeeding knowledge and attitudes. Such interventions may have a limited effect when men feel unable to discuss their knowledge and ideas with their partners. These interventions may have a dual role in empowering men to support their partners in the first weeks of breastfeeding. There may be a role to play for health professionals, such as midwives and health visitors, in ‘legitimising’ the input of men into the feeding decision process. However, this assumes that women also accept that it is legitimate for their partner to express opinion on an act which has such profound effect on them alone.

## Additional file

Additional file 1: Questions guide for interviews. The guide questions used by the lead author when interviewing participants. (DOCX 17 kb)

## References

[1] Yeung WJ, Sandberg JF, Davis-Kean PE, Hofferth SL (2004). Children’s time with fathers in intact families. J Marriage Fam.

[2] UK Government. Shared Parental Leave and Pay. 2016. https://www.gov.uk/shared-parental-leave-and-pay/. Accessed 1 Jul 2016.

[3] Faircloth F (2015). Negotiating intimacy, equality and sexuality in the transition to parenthood. Sociol Res Online.

[4] Victora CG, Bahl R, Barros AJD, França GVA, Horton S, Krasevec J (2016). For the lancet breastfeeding series group. Breastfeeding in the 21st century: epidemiology, mechanisms, and lifelong effect. Lancet.

[5] World Health Organization. Breastfeeding. 2015. http://www.who.int/topics/breastfeeding/en/. Accessed 31 Jul 2015.

[6] McAndrew F, Thompson J, Fellows L, Large A, Speed M, Renfrew MJ. Infant Feeding Survey 2010. Health and social care information Centre. 2012 https://digital.nhs.uk/media/21928/Infant-Feeding-Survey-2010-Consolidated-Report/Any/Infant-Feeding-Survey-2010-Consolidated-Report. Accessed 29 May 2015.

[7] Public Health England. Breastfeeding at 6 to 8 weeks after birth: 2015 to 2016 quarterly data. Public Health England. 2016. https://www.gov.uk/government/statistics/breastfeeding-at-6-to-8-weeks-after-birth-2015-to-2016-quarterly-data. Accessed 11th June 2016.

[8] Perez-Escamilla R, Cobas JA, Balcazar H, Benin MH (1999). Specifying the antecedents of breast-feeding duration in Peru through a structural equation model. Public Health Nutr.

[9] Vaaler ML, Castrucci BC, Parks SE, Clark J, Stagg J, Erickson T (2011). Men’s attitudes toward breastfeeding: findings from the 2007 Texas behavioral risk factor surveillance system. Matern Child Health J.

[10] MacGregor E, Hughes M (2010). Breastfeeding experiences of mothers from disadvantaged groups: a review. Community Pract.

[11] Entwistle F, Kendall S, Mead M (2012). Breastfeeding support – the importance of self-efficacy for low-income women. Matern Child Nutr..

[12] Mitchell-Box K, Braun KL, Hurwitz EL, Hayes DK (2013). Breastfeeding attitudes: association between maternal and male partner attitudes and breastfeeding intent. Breastfeed Med.

[13] Grassley JS, Spencer BS, Law BA (2012). Grandmothers’ tea: evaluation of a breastfeeding support intervention. J Perinat Educ.

[14] Craig PL, Knight J, Comino E, Webster V, Pulver LJ, Harris E (2011). Initiation and duration of breastfeeding in an aboriginal community in south western Sydney. J Hum Lact.

[15] Bristol City Council. Maternal and childrens nutrition guidelines. 2010. https://www.bristol.gov.uk/documents/20182/32995/Maternal%20and%20child%20nutrition%20guidleines%20-%20Introduction%20and%20info%20about%20Bristol.pdf/af8d084d-ad00-4265-ab0b-0d366c7a0623. Accessed 7 Sep 2015.

[16] Balsamo F, De Mari G, Maher V, Serini R, Maher V (1992). Production and pleasure: research on breast-feeding in Turin. The Anthropology of Breast-Feeding. Natural Law or Social Construct.

[17] Heck KE, Braveman P, Cubbin C, Chávez GF, Kiely JL (2006). Socioeconomic status and breastfeeding initiation among California mothers. Public Health Rep.

[18] Stewart-Knox BJ (2013). Why we don’t breastfeed our children and what we should do about it. Nutr Bull.

[19] Guttman N, Zimmerman DR (2000). (2000) low-income mothers’ views on breastfeeding. Soc Sci Med.

[20] Dowling S, Naidoo J, Pontin D, Smith PH, Hausman B, Labbok M (2012). Breastfeeding in public: Women’s bodies, women’s milk. Beyond health, beyond choice: breastfeeding constraints and realities.

[21] Kornides M, Kitsantas P (2013). Evaluation of breastfeeding promotion, support, and knowledge of benefits on breastfeeding outcomes. J Child Health Care.

[22] Maharaj N, Bandyopadhyay M (2013). Breastfeeding practices of ethnic Indian immigrant women in Melbourne. Australia Int Breastfeed J.

[23] Bandyopadhyay M (2009). Impact of ritual pollution on lactation and breastfeeding practices in rural West Bengal. India Int Breastfeed J.

[24] Zaidi F (2014). Challenges and practices in infant feeding in Islam. Br J Midwifery.

[25] Flacking R, Dykes F, Ewald U (2010). The influence of father’s socioeconomic status and paternity leave on breastfeeding duration: a population-based cohort study. Scand J Public Health.

[26] Odom EC, Li R, Scanlon KS, Perrine CG, Grummer-Strawn L (2013). Association of family and health care provider opinion on infant feeding with mother’s breastfeeding decision. J Acad Nutr Diet.

[27] Augustin AL, Donovan K, Lozano EA, Massucci DJ, Wohlgemuth F (2014). Still nursing at 6 months: a survey of breastfeeding mothers. MCN. Am J Matern Child Nurs.

[28] Van Wagenen SA, Magnusson BM, Neiger BL (2015). Attitudes towards breastfeeding among and internet panel of U.S. males aged 21-44. Matern Child Health J.

[29] Pollock CA, Bustamante-Forest R, Giarratano G (2002). Men of diverse cultures: knowledge and attitudes about breastfeeding. J Obstet Gynecol Neonatal Nurs.

[30] Arora S, McJunkin C, Wehrer J, Kuhn P (2000). Major Factors influencing breastfeeding rates: mother’s perceptions of father’s attitude and milk supply. Pediatrics.

[31] Maycock B, Binns CW, Dhaliwal S, Tohotoa J, Hauck Y, Burns S, Howat P (2013). Education and support for fathers improves breastfeeding rates: a randomized controlled trial. J Hum Lact.

[32] van Manen M (1997). Researching lived experience.

[33] Reiners GM (2012). Understanding the differences between Husserl’s (descriptive) and Heidegger’s (interpretive) phenomenological research. J Nurs Care.

[34] Earle V (2010). Phenomenology as research method or substantive metaphysics? An overview of phenomenology’s uses in nursing. Nurs Philos.

[35] Oliffe J, Mrόz L (2005). Men interviewing men about health and illness: ten lessons learned. J Mens Health Gend.

[36] Maykut P, Morehouse R (1994). Beginning qualitative research.

[37] Walls P, Parahoo K, Fleming P, McCaughan E (2010). Issues and considerations when researching sensitive issues with men: examples from a study of men and sexual health. Nurs Res.

[38] Braun V, Clarke V (2006). Using thematic analysis in psychology. Qual Res Psychol.

[39] Datta J, Graham B, Wellings K (2012). The role of father in breastfeeding: decision-making and support. Br J Midwifery..

[40] Mitchell-Box K, Braun KL (2012). Fathers’ thoughts on breastfeeding and implications for a theory-based intervention. J Obstet Gynecol Neonatal Nurs.

[41] Carlson MJ, McLanahan SS, Lamb ME (2012). Fathers in fragile families. The role of the father in child development.

[42] Sherriff N, Panton C, Hall VA (2014). New model of father support to promote breastfeeding. Community Pract..

[43] Taylor EN, Wallace LE (2012). For shame: feminism, breastfeeding advocacy, and maternal guilt. Hypatia.

[44] Wickler D (2002). Personal and social responsibility for health. Ethics Int Aff.

[45] Morrison L, Reza A, Cardines K, Foutch-Chew K, Severence C (2008). Determinants of infant-feeding choice among young women in Hilo, Hawaii. Health Care Women Int.

[46] Hoddinott P, Kroll T, Raja A, Lee AJ (2010). Seeing other women breastfeed: how vicarious experience relates to breastfeeding intention and behaviour. Matern Child Nutr.

[47] Hoddinott P, Pill R (1999). Qualitative study of decisions about infant feeding among women in east end of London. Brit Med J.

[48] Dowling S, Pontin D (2017). Using liminality to understand mothers' experiences of long-term breastfeeding: 'betwixt and between', and 'matter out of place. Health.

[49] Dowling S, Brown A (2013). An exploration of the experiences of mothers who breastfeed long-term: what are the issues and why does it matter?. Breastfeed Med.

