# Supplementary material for: ‘The mum has to live with the decision much more than the dad’; a qualitative study of men’s perceptions of their influence on breastfeeding decision-making
Source: Int Breastfeed J. 2018 Jan 16;13:3. doi: 10.1186/s13006-018-0145-1 (PMC5771101; doi:10.1186/s13006-018-0145-1)
Supplement: Additional file 1: — Questions guide for interviews. The guide questions used by the lead author when interviewing participants. (DOCX 17 kb) [file 13006_2018_145_MOESM1_ESM.docx]

Questions guide for interviews

• Can you describe to me when you first became aware that babies could feed from a woman’s breast? How did that make you feel?

• Before you became a father, or were even thinking of becoming a father, did you think breastfeeding was a normal thing to do? Why?

• Before you became a father, or were even thinking of becoming a father, did you think that men should be involved in deciding whether to breastfeed or bottle-feed? Why?

• I want you to think about the period of time leading up to the birth of your child. In what ways did you think you would feed the baby?

• Were your partner’s views the same as yours or different? How did you know that?

• Did you try and talk to your partner about feeding the baby? Why did you take that approach? How did that make you feel?

• (For men with previous children) Was that different to the situation with your previous children? Why?

• Describe to me how the feeding of the baby went when it was born? How did that make you feel?

• Did your attitude towards breastfeeding or bottle-feeding change during the months after the baby was born? Why?

• (For men with previous children) Did your attitude towards breastfeeding or bottle-feeding change after you had a previous child? Why?

• Thinking now about what you did before the child was born, would you do it differently if you had the chance? Why?
